# Implementation status of household contact tuberculosis screening by health extension workers: assessment findings from programme implementation in Tigray region, northern Ethiopia

**DOI:** 10.1186/s12913-020-4928-x

**Published:** 2020-01-31

**Authors:** Hailay Gebretnsae, Brhane Gebrekidan Ayele, Tsegay Hadgu, Esayas Haregot, Aregay Gebremedhin, Eyassu Michael, Mebrahtu Abraha, Daniel G. Datiko, Degue Jerene

**Affiliations:** 1 Tigray Health Research Institute, Mekelle, Tigray Ethiopia; 2 Tigray Regional Health Bureau, Mekelle, Tigray Ethiopia; 3 USAID/Challenge TB project, Mekelle, Tigray Ethiopia; 4 USAID/Challenge TB project, Addis Ababa, Ethiopia

**Keywords:** Tuberculosis, Tuberculosis screening, Health extension worker, Tigray, Ethiopia

## Abstract

**Background:**

In the Tigray region of Ethiopia, Health Extension Workers (HEWs) conduct Tuberculosis (TB) screening for all household (HH) contacts. However, there is limited evidence on implementation status of HH contact TB screening by HEWs. The aim of this program assessment was to describe the implementation status and associated factors of HH contact TB screening by HEWs.

**Methods:**

This programme assessment was conducted in three randomly selected districts from March to April 2018. Data was collected by using pre-tested structured questionnaire. Descriptive statistics was carried out using frequency tables. Logistic regression analysis was done to identify factors associated with HH contacts screening by HEWs.

**Results:**

In this programme assessment a total of HHs of 411 index TB cases were included. One-fifth (21.7%) of index TB cases had at least one HH contact screened for TB by HEWs. Having TB treatment supporter (TTS) during intensive phase of index TB case (AOR = 2.55, 95% CI: 1.06–6.01), health education on TB to HH contacts by HEWs (AOR = 4.28, 95% CI: 2.04–9.00), HH visit by HEWs within 6 months prior to the programme assessment (AOR = 5.84, 95% CI: 2.81–12.17) and discussions about TB activities by HEWs with Women Development Army (WDA) leaders (AOR = 9.51, 95% CI: 1.49–60.75) were significantly associated with household contact TB screening by HEWs.

**Conclusions:**

Our finding revealed that the proportion of HH contact TB screened by HEWs was low. Therefore, HEWs should routinely visit HHs of index TB cases and provide regular health education to improve contact screening practice. In addition, it is highly recommended to strengthen HEWs regular discussion about TB activities with WDA leaders and TB TTS.

## Background

Tuberculosis (TB) is a major public health problem throughout the world. Globally about 10 million people were estimated to develop TB disease in 2017 and in the same year, 558,000 people were estimated to develop TB resistant to rifampicin (RR-TB) of whom 82% had multidrug-resistant TB (MDR-TB). Ethiopia is still among the 30 high TB, TB/HIV and MDR-TB burden countries in the world, with estimated TB incidence of 164 per 100,000 populations [1]. Ethiopia has adopted” END TB strategy” [2]. Ending TB is a global priority and strategies to address interventions to achieve the targets were set. Reaching missed cases by reaching 90% of estimated TB cases including among key affected population groups is one of the key indicators [3]. Passive case finding has been the main stay to identify TB cases. However, alternative approaches including active case finding were recommended despite the cost relate to the intervention [4].

Decentralization of TB services to the community is among the key interventions to improve access to services. Which include a wide range of activities that contribute to prevention, diagnosis and treatment by community health workers and community volunteers. In 2017, globally an average of 27% notified TB cases were attributed to community based intervention [1].

Ethiopia launched the Health Extension Program (HEP) in 2003 by constructing health post staffed with trained and salaried two female Health Extension Workers (HEWs) in every kebele (the smallest administrative unit with an average population of five thousand or one thousand households) [5]. Later in 2011, Women’s Development Army (WDA) strategy was introduced to engage networks of six neighboring household (HH) women to increase efficiency of HEWs in reaching every HH in the communities. The WDA team consists of group of 30 women of HHs residing in a neighborhood in a one-to five networks (with one leader and five members) [6–8].

Community Based TB Care (CBTC) is one of HEP package of activities implemented by HEWs in the community. It includes HH contacts screening and referral of presumptive TB cases, community based Directly Observed Treatment Short course (DOTS) and follow up. The WDA team leaders support HEWs in conducting early identification and referral of presumptive TB [2]. However, the implementation of CBTC is inadequate and the national contribution of HEWs to TB cases finding is as low as 24% in 2017 [7].

The risk of acquiring and developing TB is high among HH contacts irrespective of the type of TB in the index cases [9], mainly in the first 2 years of exposure [10]. Many people with active TB do not experience typical symptoms in the early stages of the disease and may not seek care early [11]. Therefore, conducting systematic screening for active TB among HH contacts can improve early TB case finding. Early detection helps to reduce the risks of TB transmission, poor treatment outcomes, undesirable health sequelae, and adverse social and economic consequences of the disease [12]. Studies have shown that community-based systematic screening of HH contacts is feasible and can contribute to improve TB cases finding [13–16], but it is not practiced in many high burden countries [17].

In Ethiopia, HEWs are expected to conduct systematic screening for HH contacts [2]. There is limited evidence about this practice except little interventional studies conducted in southern Ethiopia [13, 16, 18]. This programme assessment will add to the pool evidence to design strategies to improve case finding to end TB. Therefore, we aimed to describe the implementation status and associated factors of HH contact screening by HEWs in Tigray region of northern Ethiopia.

## Methods

### Setting

This programme assessment was conducted in Tigray region, Northern Ethiopia in 2018. The region has seven zones which are further divided into 52 districts (34 rural and 18 urban) and 799 Kebeles (722 rural and 77 urban). It has a population of 5.5 million and 84% of these lives in rural areas. There are 712 health posts, 216 health centers and 39 hospitals that provide health services to the community. TB programme is integrated into the general health services and offered free of charge.

### Sample size

The sample size was determined by using a single population proportion formula. The computation was based on the 95% confidence interval, 5% margin of error and assuming that 50% of index TB cases whose HH contact to be screened for TB by HEWs. After 10% was added to compensate for none response rate, the final estimated sample size was 424.

### Sampling procedures

We listed all the 52 districts in region on piece of paper and randomly picked three districts using the lottery method to be included in the programme assessment. All health facilities 17 (2 primary hospitals and 15 health centers) in the randomly selected districts were included. In each selected health facility, the list of index TB cases (all forms and types) who were received treatment in the last 18 months was collected with basic information including their address from the TB log book and the index cases were sorted to each health post (operational unit for HEW) prior to data collection. Index TB cases that had no known contacts were excluded from the study. Using the listed of eligible index TB cases, the sample size was allocated proportionally based on the cases load to each health post and then the 424 index TB cases were selected by simple random sampling technique using computer based open Epi software.

### Data collection technique and data quality assurance

A structured interviewer administered questionnaire was adapted from a previous studies [19, 20] and national guideline for TBDR-TB and leprosy in Ethiopia [2]. The data was collected through face-to-face interview with index TB case or head of HH and serving HEWs. The index cases were asked about socio-demographic variables, diagnosis and treatment history, receipt of health education and HH visited by HEWs and WDA leaders, and the list of all HH members who were screened for TB symptoms-based for TB by HEWs. Their responses were verified by asking HH contacts. The head of HH or caregiver provided information about the HH’s children contact screened status by HEWs. The HEWs responsible for reaching the HHs were interviewed if they have conducted HH contact screening to confirm the practice and link their socio-demographic variables. Ten diploma nurses were trained to conducted data collection. The data collection was regularly supervised by two bachelor degree health officers. Pre-test was conducted in 5% of respondents (20 index TB cases) in a district other than the study sites. Daily data quality –check was done during the data collection period by the data collectors and supervisors.

### Operational definitions

**Index case** refers to any TB patients who were initially diagnosed with any type or form of TB. Household contact was a family member or any other person who shared the same enclosed living space with the index TB case for at least 3 months before the start of the treatment of TB case [20].

**Household contact screened for TB by HEWs** was considered if at least one household contact of index TB case that was screened for TB by HEW during home visit or at health post during treatment follows up.

### Data management and analysis

Data was entered to Epi-Info version 3.5.1 software and then exported to SPSS version 21 for analysis. Descriptive statistics was carried out using frequency tables. All variables with a *p*-value < 0.25 in bivariate analysis were analyzed using multivariable analysis model to identify predictor variables on the household contacts screened for TB by HEWs. Hosmer-Leme show goodness-of-fit was used to test the model’s fitness and the significant level was 0.78, indicating an adequate fit. The model performance was explained by 54.2% (Nagelkerke R square = 0.542). In the final model, a *p*-value < 0.05 was considered a statistically significant.

## Results

### Socio-demographic characteristics

A total 411 index TB cases were included in the study with a response rate of 96.9%. The mean age of the respondents was 40.2 (+ 15.5 Standard Deviation (SD)) years, 249(60.6%) were females, 268 (65.2%) were married, 242 (58.9%) lived in rural areas and 98 (23.8%) had no access to public transport to TB diagnostic facilities. About 233(56.7%) HHs of respondents had at least five HH members and 337(82%) HHs of respondents had an average family monthly income of < 2000.00 Ethiopan birr (74.00 USD) (Table 1).

**Table 1:** Socio-demographic characteristics of respondents in Tigray region, Northern Ethiopia, 2018(*N* = 411)

variables	Numbers	Percent
Age		
Mean age	40.2 (+ 15.52 SD) years	
Sex		
Male	162	39.4
Female	249	60.6
Marital status		
Currently in union	268	65.2
Currently not in union^a^	143	34.8
Educational status		
Illiterate	214	52.1
Literate	197	47.9
Occupation		
House wife	20	4.9
Self-employed	54	13.1
Merchant	51	12.4
Governmental employed	17	4.1
Farmer	249	60.6
Student	20	4.9
Family size		
< 4	178	43.3
5–7	171	41.6
> 8	62	15.1
Place of residence		
Urban	169	41.1
Rural	242	58.9
Availability of public transport to diagnostic facility		
Yes	313	76.2
No	98	23.8
Types of district		
Urban	67	16.3
Rule	344	83.7
Time taken to reach health post		
< 30 Minutes	315	76.6
> 30 Minutes	96	23.4
Time taken to reach health center		
< 1 h	372	90.5
> 1 h	39	9.5
Average household monthly income in US Dollar (USD)		
< 74.00 USD	337	82
> 74.00 USD	74	18

Currently not in union^a^ (single, widowed, divorce and separated)

### Index TB case diagnosis and treatment history

Of the 411 index TB cases, 247(60.1%) were diagnosed within 6–12 months before assessment and 136(33.1%) were bacteriologically confirmed pulmonary TB cases. Majority, 307 (74.7%) of index TB cases were diagnosed in public hospitals and 41(10%) of the index TB cases had history of previous treatment. From the total of index TB cases, 297(72.3%) were self-referred and 311(75.7%) were diagnosed after at least 1 month initiated symptoms. Majority of index TB cases, 324 (78.8%) had TTS during intensive phase and 317 (77.1%) during continuation phase.

### House hold contacts screening for TB and presumptive TB cases referral by HEWs

Eighty nine (21.7%) of index TB cases had at least one HH contact screened for TB by HEWs. A total of 396/1750 (22.6%) of HH contacts were screened for TB by HEWs. Of these, 49 (12.4%) were under five and, 347(87.6%) were five and above years. Most, 84.6% (*n* = 335) of the screened HH contacts were screened at their homes while 15.4% (*n* = 61) were screened at health posts. Eight (2%) presumptive TB cases were found and referred by the HEWs for further evaluation.

### HEWs’ and WDA leaders’ practices on TB prevention and control

One hundred and twenty-one (29.4%) HH members of index TB cases had received health education on TB by HEWs and 110(26.8%) HHs of index TB cases had visited by HEW 6 months prior to this programme assessment. About 12.4%(*n* = 51) of HH members of index TB cases had received health education by WDG leaders and 31.4% (*n* = 102) HH of index TB cases were visited by WDG leaders at least once per month.

### Factors associated with TB screened of house hold contacts by HEWs

Household contacts from index TB cases who had TTS were almost 3 times more likely to be screened for TB than those without TTS (AOR = 2.55; 95%CI: 1.06–6.10). Household members who received health education by HEWs (AOR = 4.28; 95%CI: 2.04–9), HHs of index TB cases which had visited by HEWs 6 months before this assessment period (AOR = 5.84; 95%CI: 2.81–12.17) and discussions about TB activities by HEWs with WDA leaders (AOR = 9.51; 95%CI: 1.49–60.75) were significantly associated with HH contacts screened for TB by HEWs (See Table 2).

**Table 2:** Logistic regression analysis of selected variables on TB screening of household contact of index TB cases by HEWs in Tigray region, Northern Ethiopia, 2018

Variables	Household contact screened for TB by HEWs		COR(95% CI)	AOR(95% CI)
	No (%)	Yes (%)		
Types of district				
Rural	277(86)	67(75.3)	1	1
Urban	45(14)	22(24.7)	2.02 (1.137–3.594*	1.24 (0.54–2.86)
Index TB case diagnosed time				
< 6 Months	74(23)	11(12.4)	1	1
6–12 Months	189(58.7)	58(65.2)	2.10 (1.027–4.150)*	2.24 (0.68–7.57)
> 12 Months	59(18.3)	20(22.5)	2.28 (1.013–5.133)*	1.70 (0.44–6.51)
Site of DOTS in intensive phase				
Primary hospital	80(24.8)	21(23.6)	1	1
Health center	218(67.7)	52(58.4)	0.91 (0.515–1.603)	0.65 (0.31–1.40)
Health post	24(7.5)	16(18)	2.54 (1.148–5.621)*	1.68(0.56–5.01)
Having treatment supporter during intensive phase				
No	75(23.3)	12(13.5)	1	1
Yes	247(76.7)	77(86.5)	1.95 (1.01–3.77)*	2.55 (1.06–6.10)*
Household members had received health education on TB by HEWs				
No	265(82.3)	25(28.1)	1	1
Yes	57(17.7)	64(71.9)	11.9 (6.91–20.50)***	4.28 (2.04–9.00)***
Household had visited by HEW in the last 6 months				
No	274(85.1)	27(30.3)	1	1
Yes	48(14.9)	62(69.7)	13.12 (7.59–22.63)***	5.84(2.81–12.17)***
Household’s mother was member of WDA team				
No	231(71.7)	51(57.3)	1	1
Yes	91(28.3)	38(42.7)	1.89 (1.16–3.078)	0.72(0.35–1.51)
Household’s index TB case visited by WDA leaders				
No	233(72.4)	34(38.2)	1	1
Don’t know/NA	12(3.7)	3(3.4)	1.71 (0.46–6.38)	2.26 (0.40–12.84)
Yes	77(23.9)	52(58.4)	4.63 (2.80–7.66)***	1.84 (0.89–43.81)
Household’s members had received health education by WDG leaders				
No	294(91.3)	66(74.2)	1	1
Yes	28(8.7)	23(25.8)	3.66 (1.98–6.75)***	1.66 (0.44–6.18)
Household’s members had screened for TB by WDA leaders				
No	302(93.8)	67(75.3)	1	1
Yes	20(6.2)	22(24.7)	4.96 (2.56–9.60)***	0.97 (0.24–3.85)
Educational status of the serving HEWs				
Certificate	79(25.9)	22(25.6)	1	1
Diploma (HEWS)	204(66.9)	44(51.2)	0.78 (0.436–1.375)	1.18(0.47–2.95)
Diploma (Nurse)	22(7.2)	20(23.3)	3.26 (1.514–7.037)***	5.18(0.80–33.60)
HEWs had discussions with WDA leaders on TB activities				
No	44(14.4)	2(2.3)	1	1
Yes	261(85.6)	84(97.7)	7.1(1.681–29.831)**	9.51(1.49–60.75)*
Availability of recording tools in the serving health posts				
Poor	134(43.9)	27(31.4)	1	1
Fair	171(56.1)	59(68.6)	1.71(1.03–2.847)*	1.84(0.75–4.52)

*statistically significant at 0.05 < *p* < 0.01, ** statistically significant at 0.01 < *p* < 0.001, *** statistically significant at *p* < 0.001

Variables with *P*-value < 0.25 in bivariate analysis were entered in the final model (Time taken to reach health facility, household average monthly income, family size, knowledge of the respondents on TB, type of index TB case, index TB case currently on treatment, marital status of the serving HEW, involvement of Kebeles administrator on TB program and availability of job aid in the serving health post)

## Discussion

This programme assessment revealed that HH contact TB screened by HEWs was low. Only one-fifth (21.7%) of index TB cases had at least one HH contact screened for TB by HEWs. The current result is far behind to achieve the national TB screening program implementation wherein HEWs have been given the responsibility to screen all HH contacts in Ethiopia [21], and WHO target which at least 90% of HH contacts of index TB cases should be screened for TB [1].

Our finding is slightly higher than reported from India 14% of household contacts were screened for TB by health care workers [22]. However, it is lower than the reported of index TB cases had at least one HH contact screened for TB ranged from 33.7 to 79% in Ethiopia [16, 18, 20, 23], and 30.7% in Vietnam [24]. The discrepancy could be due to the difference of TB screening approaches and studies methods.

The low TB screening practice of HH contacts could be due to the low HH visits by HEWs. Our findings show that HH contacts whose houses had visited by HEWs were 6 times more likely to be screened for TB than those houses which had not visited by HEWs within the last 6 months. This finding is supported with different studies which recommended for a strong follow up including household level visits by HEWs for a better TB case detection [13, 16, 19, 25–27].

Health education is an instrument to create awareness about TB and health seeking. HH contacts who had received health education on TB by HEWs were 4 times more likely to be screened by HEWs for TB than those who had not. Studies showed that demand of TB screening HH contacts can be improved by increasing awareness about TB through health education [28], and making the HH contacts to seek screening service at health posts by HEWs [29]. Provision of community health education was also associated with higher presumptive TB case referral [19, 30].

Patient support mechanism enhances adherence to treatment. This is done through health education at treatment site and linking with TTS at the community. HH contacts of index TB cases those who had TB TTS were nearly 3 times more likely to be screened for TB than those without TTS. This might be due to TTS play a role in improving the knowledge of the index cases [31–33]. It is also possible that the level of knowledge of HH members and TTS could enhance the capacity of the HH members to be screened or capacity of TTS to create awareness and screen respectively. They may also contribute in improving the knowledge and health seeking behavior of the HHs contacts. Additionally, as TTS could have the chance to visit both the HH of the index case (for community DOTS) and the health posts (to collect the drug), this may make them as a bridge between the index TB case and the HEWs which results in HH contact screening for TB by HEWs.

Our finding has come up with a result that indicates HEWs who discussed TB issues through regular meeting with WDG leaders had a role in improving TB screening practice of HH contacts of index TB cases. This result indicated those HH contacts of index TB cases whose WDA leaders had discussion on TB related issues through regular meeting with HEWs were 9 times more likely to be screened for TB by HEWs than those who had not. This could be a result of WDA leaders who have regular meeting and discussion with HEWs might further created awareness on HH contacts about TB and improved their health seeking behavior for TB screening. Contribution of such active participation of community members were also revealed in other related studies [27, 34–37].

### Limitation of the study

Failure to recall about previous screening by HEWs might introduce recall bias and underestimate the screening practice. Additionally social desirability bias may be introduced that takes in the form of over-reporting for those opinions socially acceptable and under-reporting for those socially undesirable. Another limitation is that our study did not have any control areas or regions for comparison.

## Conclusion

Our finding showed that the HH of index TB cases with at least one HH contact screened for TB by HEWs was low only 21.7%. Therefore, HEWs should routinely visits HHs of index TB cases and regular provision of health education to HH contacts on TB to improve TB screening practice. In addition, it is highly recommended to strengthen HEWs regular discussion about TB activities with WDA leaders and TB TTS.

## Data Availability

The datasets used and/or analyzed during the current study are available from the corresponding author on reasonable request.

## Abbreviations

AOR: Adjusted odds ratio
CI: Confidence interval
DOTS: Directly observed treatment short course
HEW: Health extension worker
HH: Household
TB: Tuberculosis
WDA: Women Development Army

## References

[1] World Health Organization (WHO) (2018). Global Tuberculosis Report 2018.

[2] Federal Democratic Republic of Ethiopia Ministry of Health (2017). National Guideline for TBDR-TB and leprosy in Ethiopia sixth ed.

[3] Herbert N, Masham BS, Suttie BA, Sharma V, Albani S, Domenti O (2018). Advancing political will to end the tuberculosis epidemic. Lancet Infect Dis.

[4] Harries AD, Lin Y, Kumar AMV, Satyanarayana S, Takarinda KC, Dlodlo RA (2018). What can National TB Control Programmes in low- and middle-income countries do to end tuberculosis by 2030?.

[5] Federal Democratic Republic of Ethiopia Ministry of Health (2005). Health Sector Strategic Plan (HSDP-III) 2005/6–2009/10.

[6] Federal Democratic Republic of Ethiopia Ministry of Health (2015). Health Sector Transformation Plan (HSTP) 2015/16–2019/20 (2008–2012 EFY).

[7] Federal Democratic Republic of Ethiopia Ministry of Health (2017). Annual Health Sector Performance Report EFY 2009 (2016/17).

[8] Federal Democratic Republic of Ethiopia Ministry of Health. Policy and practice Information for Action. Q Health Bull. 2013;5(1):4.

[9] Gashu ZJD, Ensermu M, Habte D, Melese M, Hiruy N (2016). The Yield of Community-Based “Retrospective” Tuberculosis Contact Investigation in a High Burden Setting in Ethiopia. PLoS One.

[10] Fox GJBS, Britton WJ, Marks GB (2013). Contact investigation for tuberculosis: a systematic review and meta-analysis. Eur Respir J.

[11] World Health Organization (WHO) (2014). The End TB Strategy, Global strategy and targets for tuberculosis prevention, care and control after 2015.

[12] World Health Organization (WHO) (2013). Systematic screening for active tuberculosis: principles and recommendations.

[13] Yassin MA, Datiko DG, Tulloch O, Markos P, Aschalew M (2013). Innovative Community-Based Approaches Doubled Tuberculosis Case Notification and Improve Treatment Outcome in Southern Ethiopia. PLoS One.

[14] Oshi DC, Omeje JC, Oshi SN, Alobu IN, Chukwu NE, Nwokocha C (2017). An evaluation of innovative community based approaches and systematic tuberculosis screening to improve tuberculosis case detection in Ebonyi state, Nigeria. Int J Mycobacteriol.

[15] McAllister S, Wiem Lestari B, Sujatmiko B, Siregar A, Sihaloho ED, Fathania D (2017). Feasibility of two active case finding approaches for detection of tuberculosis in Bandung City, Indonesia. Int Union Against Tuberc Lung Dis.

[16] Datiko DG, Yassin MA, Theobald SJ, Blok L, Suvanand S, Creswell J (2017). Health extension workers improve tuberculosis case finding and treatment outcome in Ethiopia: a large-scale implementation study. BMJ Glob Health.

[17] Blok L, Sahu S, Creswell J, Alba S, Stevens R, Bakker MI (2015). Comparative meta-analysis of tuberculosis contact investigation interventions in eleven high burden countries. PLoS One.

[18] Datiko DG, Yassin MA, Theobald SJ, Cuevas LE (2017). A community-based isoniazid preventive therapy for the prevention of childhood tuberculosis in Ethiopia. Int J Tuberc Lung Dis.

[19] Getnet F, Hashi A, Mohamud S, Mowlid H, Klinkenberg E (2017). Low contribution of health extension workers in identification of persons with presumptive pulmonary tuberculosis in Ethiopian Somali Region pastoralists. BMC Health Serv Res.

[20] Gebregergs GB, Alemu WG (2015). Household Contact Screening Adherence among Tuberculosis Patients in Northern Ethiopia. PLoS One.

[21] Federal Democratic Republic of Ethiopia Ministry of Health (2017). Comprehensive training manual for clinical and programmatic management of TBL and TB/HIV.

[22] Banu Rekha VV, Jagarajamma K, Wares F, Chandrasekaran V, Swaminathan S (2009). Contact screening and chemoprophylaxis in India’s Revised Tuberculosis Control Programme: a situational analysis. Int J Tuberc Lung Dis.

[23] Ramos JM, Biru D, Tesfamariam A, Reyes F, Górgolas M (2013). Screening for tuberculosis in family and household contacts in a rural area in Ethiopia over a 20-month period. Int J Mycobacteriol.

[24] Thanh (2014). A household survey on screening practices of household contacts of smear positive tuberculosis patients in Vietnam. BMC Public Health.

[25] Datiko DG, Lindtjørn B (2009). Health Extension Workers Improve Tuberculosis Case Detection and Treatment Success in Southern Ethiopia: A Community Randomized Trial. PLoS One.

[26] Pothukuchi M, Nagaraja SB, Kelamane S, Satyanarayana S, Shashidhar BS (2011). Tuberculosis Contact Screening and Isoniazid Preventive Therapy in a South Indian District: Operational Issues for Programmatic Consideration. PLoS One.

[27] Ahmad QG, Rashidi MK (2018). Community interventions to improve access to TB services in Afghanistan. Technical brief USAID/MSH, Challenge TB.

[28] Ekwueme (2014). strengthening contact tracing capacity of pulmonary tuberculosis patients in Enugu, southeast Nigeria: a targeted and focused health education intervention study. BMC Public Health.

[29] Yitayal (2014). Health extension program factors, frequency of household visits and being model households, improved utilization of basic health services in Ethiopia. BMC Health Serv Res.

[30] Tulloch O, Theobald S, Morishita F, Datiko DG, Asnake G, Tesema T (2015). Patient and community experiences of tuberculosis diagnosis and care within a community-based intervention in Ethiopia: a qualitative study. BMC Public Health.

[31] Zhang H, Ehiri J, Yang H, Tang S, Li Y (2016). Impact of Community-Based DOTon Tuberculosis Treatment Outcomes: A Systematic Review and Meta-Analysis. PLoS One.

[32] Ong’ang’o JR, Mwachari C, Kipruto H, Karanja S (2014). The Effects on Tuberculosis Treatment Adherence from Utilising Community Health Workers: A Comparison of Selected Rural and Urban Settings in Keny. PLoS One.

[33] Soomro MH, Qadeer E, Khan MA, Morkve O (2012). Treatment supporters and their impact on treatment outcomes in routine tuberculosis program conditions in Rawalpindi District, Pakistan. Tanaffos.

[34] Uwimana J, Zarowsky C, Hausler H, Swanevelder S, Tabana H, Jackson D (2013). Community-based intervention to enhance provision of integrated TB-HIV and PMTCT services in South Africa. Int J Tuberc Lung Dis.

[35] Rachlis B, Naanyu V, Wachira J, Genberg B, Koech B, Kamene R (2016). Community Perceptions of Community Health Workers (CHWs) and Their Roles in Management for HIV, Tuberculosis and Hypertension in Western Kenya. PLoS One.

[36] le Roux K, le Roux I, Mbewu N, Davis E (2015). The role of community health workers in the re-engineering of primary health care in rural Eastern Cape. South Afr Fam Pract.

[37] Adejumo AO, Azuogu B, Okorie O, Lawal OM, Onazi OJ, Gidado M (2016). Community referral for presumptive TB in Nigeria: a comparison of four models of active case finding. BMC Public Health.

